# Multidirectional wall shear stress is associated with thrombotic risk in isolated coronary artery ectasia

**DOI:** 10.3389/fcvm.2025.1705263

**Published:** 2025-11-18

**Authors:** Jiejun Sun, Muyun Tang, Shitong Liu, Zhenyu Liu, Zhujun Shen, Hongzhi Xie, Wei Wu, Hao Qian, Liang Wang, Zhiyu Zhang, Ran Tian, Shuyang Zhang

**Affiliations:** 1 Department of Cardiology, Peking Union Medical College Hospital, Chinese Academy of Medical Sciences & Peking Union Medical College, Beijing; 2 Department of Medical Research Center, State Key Laboratory of Complex Severe and Rare Diseases, Peking Union Medical College Hospital, Chinese Academy of Medical Science and Peking Union Medical College, Beijing, China; 3 Department of Research and Development, Beijing Uninsim Tech Co. Ltd, Beijing, China

**Keywords:** isolated coronary artery ectasia, hemodynamic changes, computational fluid dynamics, wall shear stress, thrombosis

## Abstract

**Background:**

Thrombosis is a critical and lethal complication of isolated coronary artery ectasia (iCAE), potentially linked to hemodynamic changes, particularly wall shear stress (WSS) alterations. However, the association between thrombosis and multidirectionality of blood flow, and thus of WSS, is not well defined.

**Methods:**

This study retrospectively enrolled 34 patients diagnosed with iCAE. In total, 53 ectatic coronary arteries were identified for hemodynamics analysis, of which 11 had thrombosis and 42 did not. We used an automated workstation for coronary three-dimensional reconstruction and conducted computational fluid dynamics (CFD) analysis. Particularly, we analyzed the mean changes in the following multidirectional WSS metrics at the ectatic segments: time-averaged WSS (TAWSS), oscillatory shear index (OSI), relative residence time (RRT), transverse WSS (transWSS), cross-flow index (CFI), and topological shear variation index (TSVI).

**Results:**

Local TAWSS_mean_, OSI_mean_, RRT_mean_, transWSS_mean_, CFI_mean_, and TSVI_mean_ of iCAE with thrombosis were significantly higher than those of iCAE without thrombosis (*p* < 0.05 for all). Logistic regression analyses indicated that RRT_mean_ (OR = 1.010, 95% CI: 1.002–1.019, *p* = 0.021) and transWSS_mean_ (OR = 1.992, 95% CI: 1.257–2.939, *p* = 0.003) were independently associated with the risk of thrombosis among patients with iCAE. The RRT_mean_ + transWSS_mean_ model [Area Under the Curve (AUC) = 0.911] exhibited a greater capacity for identifying the risk of thrombosis among patients with iCAE compared to maximum vascular diameter (MVD) (AUC = 0.789, *p* = 0.048).

**Conclusions:**

Multidirectional WSS metrics can help identify iCAE with a higher risk of thrombosis. It can improve thrombosis risk stratification.

## 1 Introduction

Isolated coronary artery ectasia (iCAE) is a rare disease characterized by pathological dilatation of the coronary artery exceeding 1.5-fold the diameter of the adjacent normal segment and the absence of significant atherosclerotic narrowing (1, 2). Compared to the similarly defined term coronary artery aneurysm (CAA), iCAE refers to more diffuse lesions (3, 4). Previous studies have indicated that CAE may be a high-risk lesion due to the abnormal dilation and impaired coronary flow, forming an environment susceptible to thrombus and acute coronary events (5, 6). Given the largely unknown natural course of iCAE, its management presents significant challenges. The risk of thrombosis and the necessity for antithrombotic therapy in this condition remain subjects of ongoing investigation (2, 7). So far, the maximum vascular diameter (MVD) has been regarded as the important basis for assessing the risk of thrombosis among individuals with iCAE and deciding the anti-thrombotic strategy, referring to the guidelines on CAA due to Kawasaki disease (8, 9). However, multiple studies have suggested that the risk of thrombosis among patients with iCAE appears to be more closely associated with hemodynamic alterations than with MVD (1, 10, 11).

Patient-specific hemodynamics analysis has become feasible with the advancement of computational fluid dynamics (CFD) technology. CFD enables the computation of hemodynamic metrics such as wall shear stress (WSS)/time-averaged wall shear stress (TAWSS), oscillatory shear index (OSI), and relative residence time (RRT) with high accuracy, and certain levels of these hemodynamic metrics can induce platelet activation, resulting in thrombosis (12–14). To date, there are no CFD-related studies on iCAE. Gutierrez et al. (11, 15) performed two small-sized studies to explore hemodynamic changes of CAA due to Kawasaki disease. Blood flow simulations showed that the hemodynamic metrics could highly differ in cases with same MVD. Furthermore, these metrics may provide improved thrombotic risk stratification compared to current MVD-based metrics. It's worth noting that in these studies, each CAA was treated as a discrete case, and they did not comprehensively assess the hemodynamic parameters of the “entire” coronary artery. Moreover, since two studies only use TAWSS, OSI, and RRT as predictors of impaired blood flow, the multidirectionality of blood flow, induced by its pulsatile nature and three-dimensional geometry, is not fully taken into account. Recently, new WSS metrics have been developed to capture this multidirectional flow behavior, including topological shear variation index (TSVI) (16), transverse WSS (transWSS) (17), and its normalized version known as the cross-flow index (CFI) (18). These new WSS metrics have not been investigated before in studies on iCAE and CAA.

Therefore, this study aimed to compare alterations in six distinct (multidirectional) WSS metrics between iCAE with and without thrombosis and identify key hemodynamic parameters associated with the risk of thrombosis in iCAE.

## 2 Methods

### 2.1 Study population

From July 2007 to July 2023, patients diagnosed with iCAE by coronary angiography at the Peking Union Medical College Hospital, Beijing, China were included in this study. Written informed consent was obtained from each patient included in the study, the study protocol conforms to 1975 Declaration of Helsinki and its later amendments and has been priorly approved by the the Ethics Committee of Peking Union Medical College Hospital (Ethics batch number: I-22PJ893). The exclusion criteria were as follows: (i) coronary artery stenosis >20%, or a history of prior percutaneous coronary intervention or coronary artery bypass grafting; (ii) thrombotic ectatic coronaries without complete recanalization; (iii) concurrent severe heart disease (e.g., heart failure, malignant arrhythmia, and severe valvular disease) or hemodynamic instability; (iv) Long-term use of antithrombotic drugs (including antiplatelet therapy and anticoagulation therapy) before thrombotic events occur in ectatic coronary arteries; (v) poor clarity of coronary angiography, absence of suitable sites for three-dimensional coronary reconstruction, or software recognition failure; (vi) Stenosis or ectasia of the coronary artery openings, which may influence the precision of multidirectional WSS metrics calculations. We finally enrolled 34 patients diagnosed with iCAE. In total, 53 ectatic coronary arteries were identified for hemodynamics analysis, of which 11 had thrombosis and 42 did not. The baseline clinical characteristics of the enrolled patients are provided in Supplementary Table S1.

### 2.2 Coronary three-dimensional reconstruction

**Figure 1:**
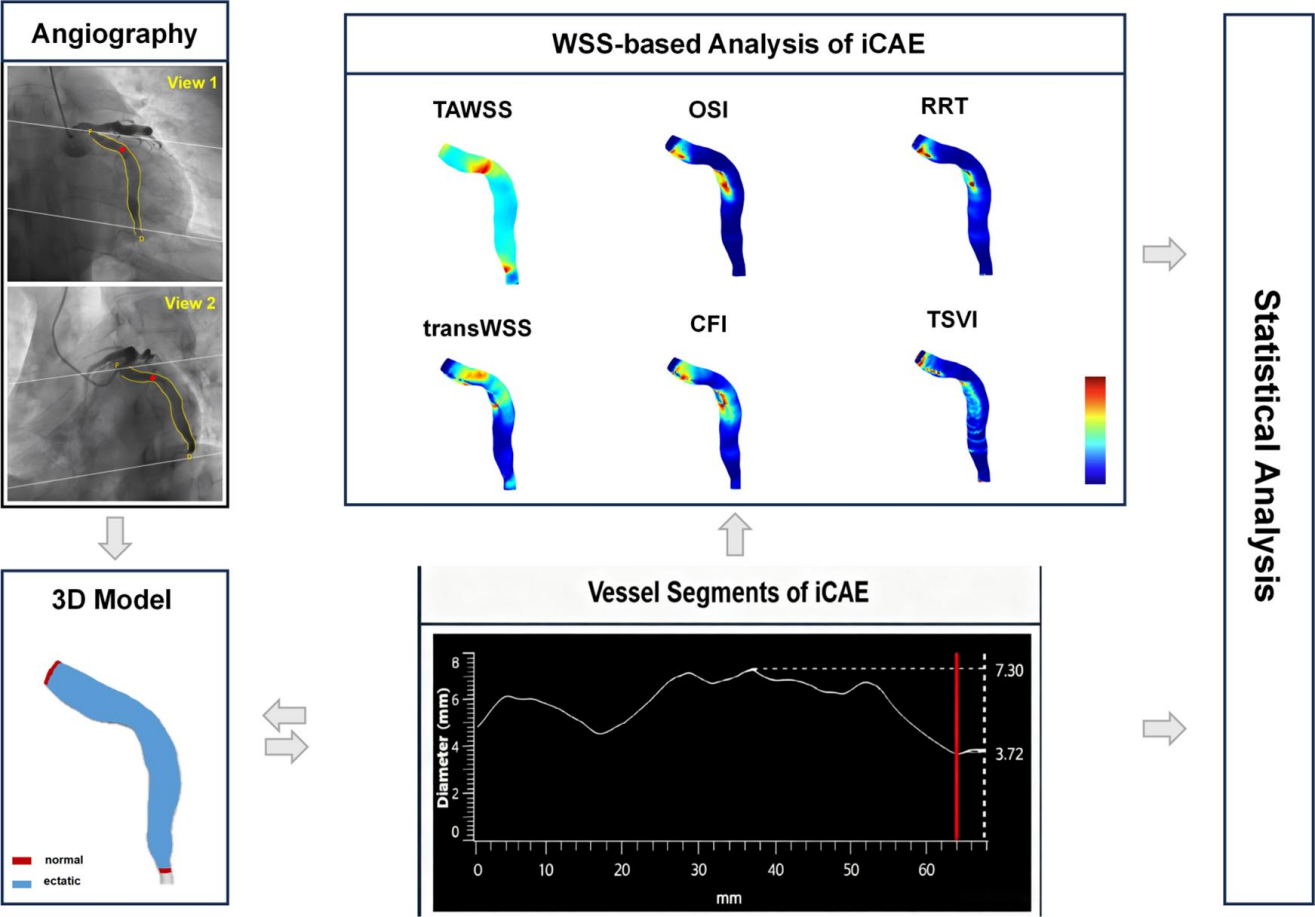
Workflow of the study. iCAE, isolated coronary artery ectasia; TAWSS, time-averaged wall shear stress; OSI, oscillatory shear index; RRT, relative residence time; transWSS, transverse wall shear stress; CFI, cross-flow index; TSVI, topological shear variation index.

The workflow of the study is shown in Figure 1. We conducted three-dimensional coronary reconstruction using the CAAS Workstation WSS software 8.2 prototype (Pie Medical Imaging, Maastricht, and the Netherlands). Its accuracy and reproducibility have been validated in other publications (19–21). DICOM files for three-dimensional reconstruction of coronary angiography images contained information on rotation, angulation, source image distance, and pixel size on the detector. First, two angiographic end-diastolic images with a difference in spatial angle of more than 30 degrees were identified by the software. Second, automated lumen contour detection was enabled and manually corrected when needed. Spatial alignment between the angiographic projection is automatically performed by identifying a “common image point”, the red dot in the two-dimensional segmentation, which is located at the same anatomical position. The three-dimensional reconstruction of the coronary artery commenced at the coronary opening and encompassed the entire main branch and branches with a diameter ≥2 mm if feasible, in addition to the ectatic segments.

### 2.3 Computational simulations

Based on three-dimensional coronary reconstruction, CFD simulations were carried out automatically by CAAS Workstation WSS software 8.2 prototype. Adopting a radius-based strategy and incorporating five near-wall tetrahedral layers, the fluid domain was meshed with P1-P1 tetrahedral elements using NETGEN (22). The governing equations of fluid motion were solved in their discretized form under unsteady-state conditions by applying the finite element code Kratos (23). Blood was modeled as a homogeneous, incompressible, Newtonian fluid with a density of 1060 kg/m³ and a dynamic viscosity of 0.0035 Pa·s. Rigid vessel walls were assumed, with no-slip conditions applied at the wall boundaries. Following a previously described scaling method (24), inlet boundary conditions were defined using generic Doppler velocity profiles, which were customized to each patient based on the individual inlet cross-sectional diameter. Inlet flow rates were set using parabolic velocity profiles, while zero pressure was applied at the outlets (25).

### 2.4 Multidirectional WSS metrics

The quantitative characterization of endothelial shear forces included the following multidirectional WSS metrics: TAWSS, OSI, RRT, transWSS, CFI, and TSVI. TAWSS denotes the average WSS throughout a cardiac cycle, reflecting the mean force exerted by blood flow on the vascular wall and serving as a key metric for evaluating hemodynamic impact (26). OSI, dimensionless, gauges directional WSS fluctuations, with greater values suggesting increased vortex and reverse flow (27). RRT, also dimensionless, is associated with the blood's average dwell time near the vascular wall to the cardiac cycle's duration, with higher values indicating slower flow and prolonged dwell times (28). TransWSS quantifies the WSS vector component normal to blood flow's primar*y* axis, reflecting lateral wall friction. It is a critical marker for assessing hemodynamic disturbance (17). CFI, the ratio of transWSS to TAWSS, assesses lateral flow intensity, and higher values indicate substantial lateral flow and potential turbulence (18). TSVI quantifies the variability of the local contraction/expansion motion's exerted by WSS on the endothelium along the cardiac cycle, with higher values denoting more complex hemodynamic conditions (16).

The calculation formulas for multidirectional WSS metrics are presented in Supplementary Table S2. Hemodynamic parameters were automatically computed using the aforementioned software. The mean, maximum, and minimum values of the hemodynamic parameters in the ectatic coronary segments were recorded. Utilizing coronary angiography images and coronary vessel diameter curves (supplemented by intravascular imaging if necessary), coronary segments are categorized into normal segments and ectatic segments. The focus of hemodynamic analysis is confined to ectatic segments (Figure 1). The susceptibility of maximum and minimum values to errors in three-dimensional vascular reconstruction and blood flow simulation can lead to significant variability in results; thus, we focused on the mean values of hemodynamic parameters (19, 29).

### 2.5 Statistical methods

Statistical analyses were conducted using SPSS (version 26.0) and R software (version 4.3.1). The normality of data was assessed using the Shapiro–Wilk test and Histogram. Normally distributed continuous data were expressed as mean ± SD, while non-normally distributed data were reported as median (Q1–Q3). Categorical variables were expressed as numerals (percentages) and evaluated using the chi-square test. Continuous variables with normal distribution were compared using the two independent-sample two independent-sample t-test and continuous variables without normal distribution were compared using the Mann–Whitney *U* test. Both univariable and multivariable logistic regression analyses were conducted to ascertain predictors of thrombotic events in iCAE. Initially, potential risk factors of thrombosis were identified through univariable analysis. Subsequently, variables with a *p*-value of less than 0.10 were subjected to a “forward, conditional” multivariable regression model. The predictive accuracy of the model was determined using the area under the receiver operating characteristic (ROC) curve. The area under the curve (AUC) was compared using the Bootstrap method. All tests were two-tailed, with statistical significance defined as *p* *<* 0.05.

## 3 Results

### 3.1 Imaging characteristics of ectatic coronary arteries

**Figure 2:**
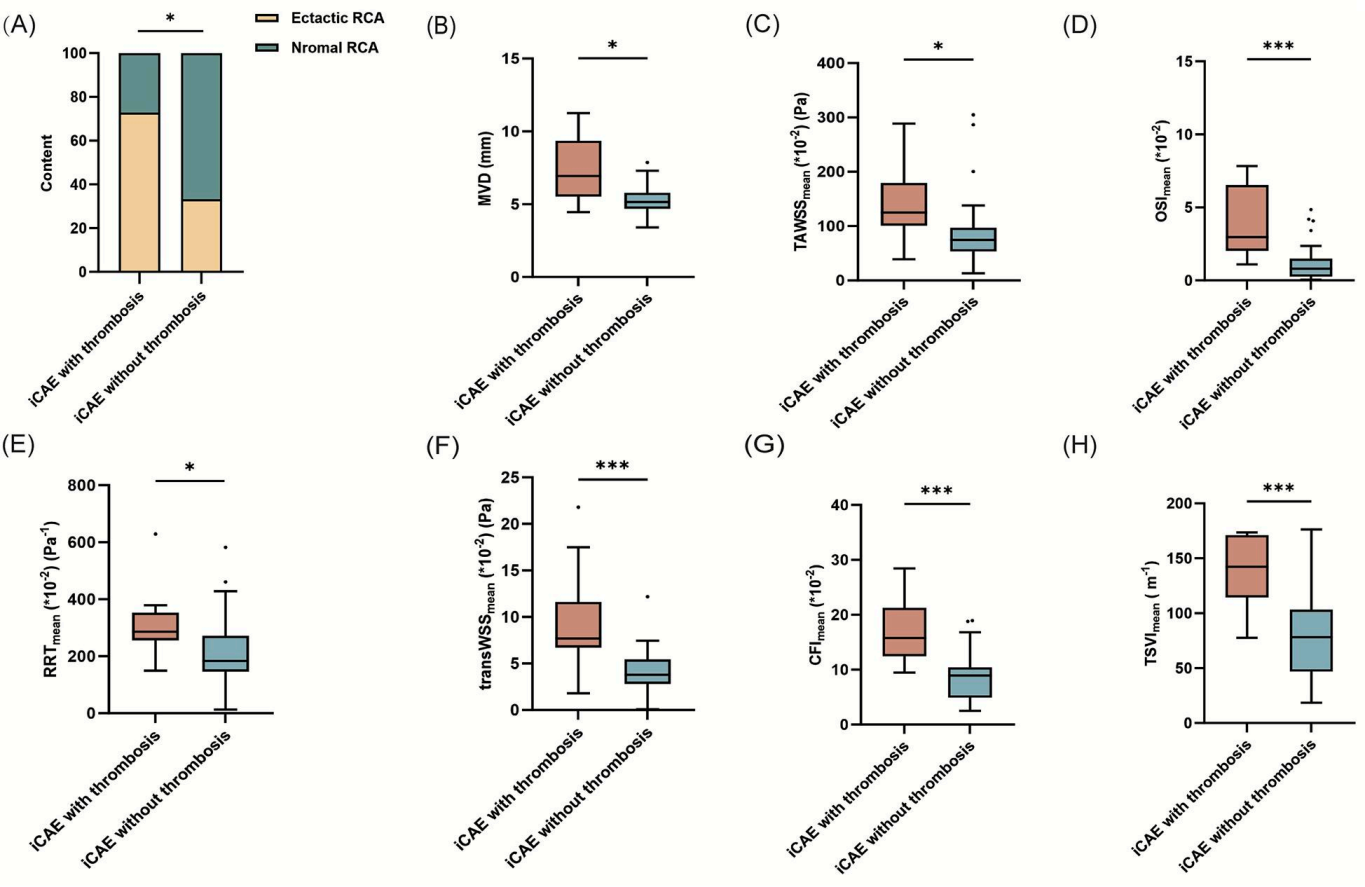
Comparison of the mean values of hemodynamic parameters between iCAE with and without thrombosis. *p < 0.05; **p < 0.01; ***p < 0.001; iCAE, isolated coronary artery ectasia; RCA, right coronary artery; MVD, maximum vascular diameter; TAWSS, time-averaged wall shear stress; OSI, oscillatory shear index; RRT, relative residence time; transWSS, transverse wall shear stress; CFI, cross-flow index; TSVI, topological shear variation index.

The imaging characteristics of the 53 ectatic coronary arteries are presented in Table 1. RCA was the most frequently affected artery (41.5%), followed by the left circumflex artery (LCX, 34.0%). The left anterior descending artery (LAD) was the least affected (24.5%) artery. iCAE with thrombosis exhibited a higher proportion of RCA ectasia compared to iCAE without thrombosis (72.7% vs. 33.3%, *p* = 0.044) (Figure 2A). Diffuse ectatic lesions were common in iCAE (75.5%), and there was no significant difference between the two groups (81.8% vs. 73.8%, *p* = 0.876). Notably, MVD in the iCAE with thrombosis was significantly larger than that in iCAE without thrombosis (7.2 ± 0.7 vs. 5.3 ± 0.1 mm, *p* = 0.015) (Figure 2B).

**Table 1:** Imaging and hemodynamic characteristics of ectatic coronary arteries.

Anatomical and hemodynamic parameters		iCAE with thrombosis (*n* = 11)	iCAE without thrombosis (*n* = 42)	*p* value
Ectatic LAD		2 (18.2)	11 (26.2)	0.867
Ectatic LCX		1 (9.1)	17 (40.5)	0.110
Ectatic RCA		8 (72.7)	14 (33.3)	0.044
Diffuse ectatic lesion^a^		9 (81.8)	31 (73.8)	0.876
MVD (mm)		7.2 ± 0.7	5.3 ± 0.1	0.015
Multidirectional shear stress metrics				
TAWSS (*10^−2^) (Pa)	Mean	124.9 (100.9–179.2)	74.6 (53.5–96.8)	0.013
	Maximum	700.0 (274.7–1,095.8)	235.1 (134.6–362.2)	0.003
	Minimum	9.9 (7.5–15.3)	15.0 (11.4–18.2)	0.007
OSI (*10^−2^)	Mean	3.0 (2.0–6.5)	0.8 (0.3–1.5)	<0.001
	Maximum	34.2 (31.7–40.1)	15.5 (4.8–29.0)	<0.001
	Minimum	0 (0.001–0.004)	0 (0–0.001)	0.004
RRT (*10^−2^) (Pa^−1^)	Mean	286.3 (256.2–353.0)	183.4 (146.2–271.8)	0.011
	Maximum	4,712.9 (2,322.4–6,369.8)	1,349.0 (747.7–2,887.6)	<0.001
	Minimum	14.4 (9.2–36.5)	45.0 (30.1–69.7)	0.006
transWSS (*10^−2^) (Pa)	Mean	7.7 (6.7–11.6)	3.8 (2.8–5.4)	<0.001
	Maximum	46.3 (25.3–60.7)	12.2 (10.2–17.3)	<0.001
	Minimum	0.4 (0.2–0.9)	0.2 (0.1–0.3)	0.004
CFI (*10^−2^)	Mean	15.8 (12.5–21.3)	9.0 (4.9–10.4)	<0.001
	Maximum	66.9 ± 2.6	42.3 ± 3.0	<0.001
	Minimum	0.6 (0.5–0.7)	0.2 (0.2–0.4)	0.007
TSVI (m^−1^)	Mean	142.2 (114.5–171.1)	78.2 (46.9–103.3)	<0.001
	Maximum	1,191.4 ± 81.6	778.6 ± 62.4	0.001
	Minimum	4.9 (3.4–7.4)	2.7 (1.7–4.0)	0.001

iCAE, isolated coronary artery ectasia; LAD, left anterior descending artery; LCX, left circumflex artery; RCA, right coronary artery; MVD, maximum vascular diameter; TAWSS, time-averaged wall shear stress; OSI, oscillatory shear index; RRT, relative residence time; transWSS, transverse wall shear stress; CFI, cross-flow index; TSVI, topological shear variation index.

a Diffuse ectatic lesion was defined as those in which the ectatic segment extends beyond one-third of the arterial length.

### 3.2 Multidirectional WSS metrics can help identify risk of thrombosis

Quantitative analysis of multidirectional WSS metrics exhibited significant differences between iCAE with thrombosis and iCAE without thrombosis (Table 1). TAWSS_mean_ of ectatic segments was significantly higher in iCAE with thrombosis than in iCAE without thrombosis [124.9 (100.9–179.2) vs. 74.6 (53.5, 96.8) *10^−2^ (Pa), *p* = 0.013]. Similarly, local OSI_mean_ [(3.0 (2.0, 6.5) vs. 0.8 (0.3, 1.5)*10^−2^, *p* < 0.001], RRT_mean_ [286.3 (256.2, 353.0) vs. 183.4 (146.2, 271.8) *10^−2^ (Pa^−1^), *p* = 0.011], transWSS_mean_ [7.7 (6.7, 11.6) vs. 3.8 (2.8, 5.4)*10^−2^ (Pa), *p* < 0.001], CFI_mean_ [15.8 (12.5, 21.3) vs. 9.0 (4.9, 10.4)*10^−2^, *p* < 0.001] and TSVI_mean_ [142.2 (114.5, 171.1) vs. 78.2 (46.9, 103.3) (m^−1^), *p* < 0.001] were significantly higher in iCAE with thrombosis than in iCAE without thrombosis (Figures 2C–H). Furthermore, the maximum values of all multidirectional WSS metrics between the two groups not only revealed the aforementioned trend but also exhibited significant differences (Table 1, Supplementary Figures S1A–F). The minimum values of the majority of the multidirectional WSS metrics were significantly higher in iCAE with thrombosis than in iCAE without thrombosis, except for TAWSS and RRT (Table 1, Supplementary Figures S2A–F).

**Figure 3:**
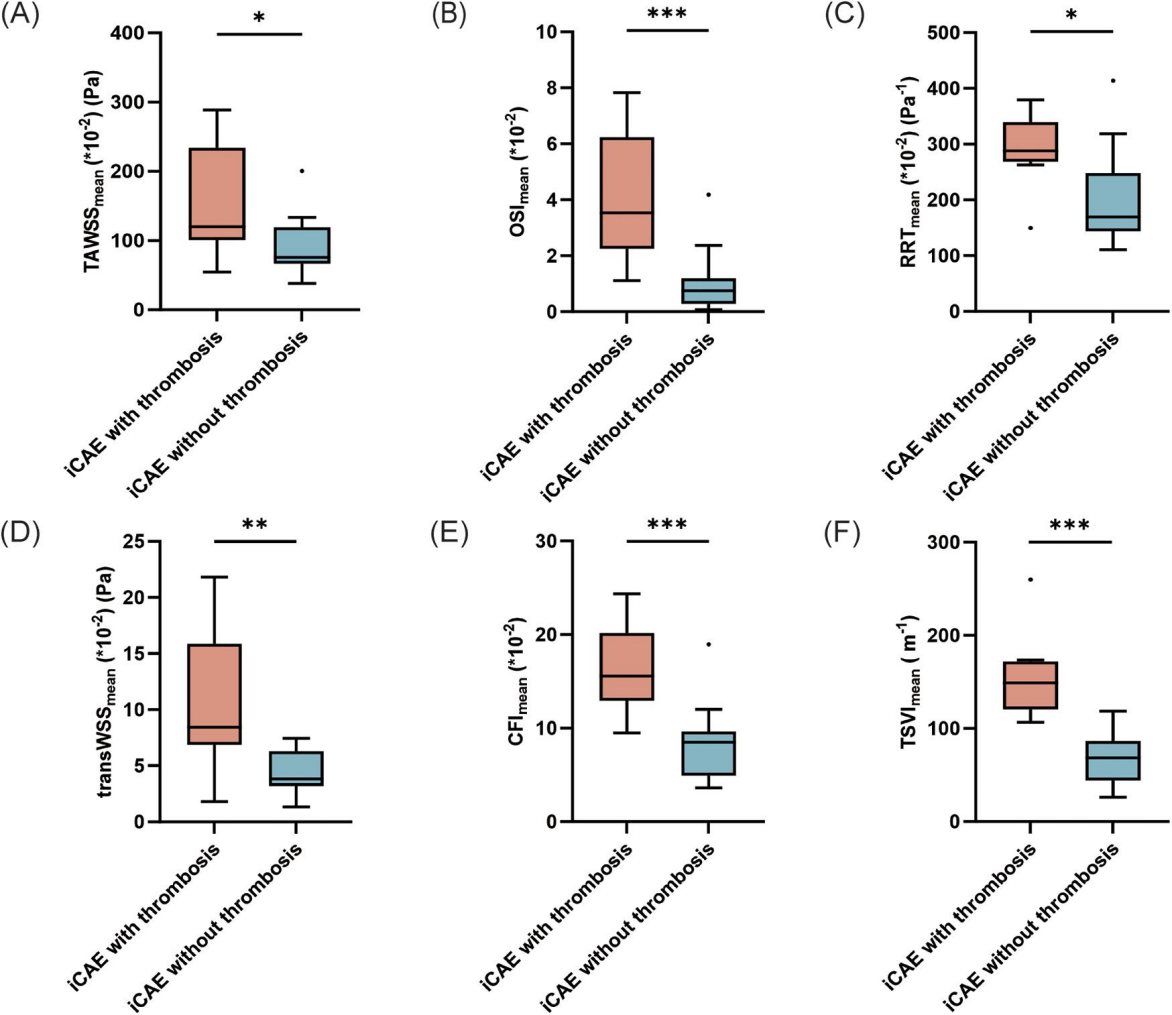
Comparison of the mean values of hemodynamic parameters between ectatic RCA with and without thrombosis. *p < 0.05; **p < 0.01; ***p < 0.001. iCAE, isolated coronary artery ectasia; TAWSS, time-averaged wall shear stress; OSI, oscillatory shear index; RRT, relative residence time; transWSS, transverse wall shear stress; CFI, cross-flow index; TSVI, topological shear variation index.

**Figure 4:**
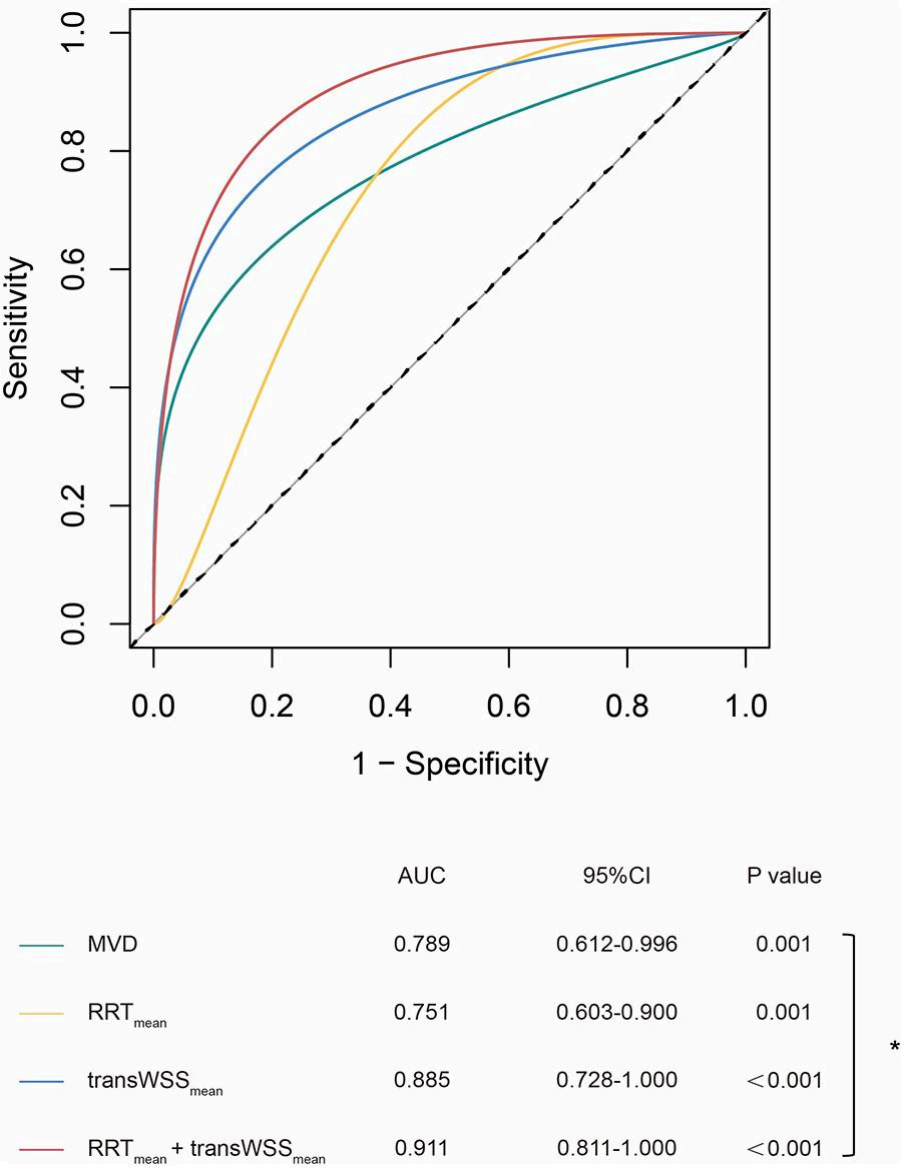
ROC curves of geometric and hemodynamic parameters in predicting thrombosis. *p < 0.05; MVD, maximum vascular diameter; RRT, relative residence time; transWSS, transverse wall shear stress.

Subgroup analysis was conducted on ectatic RCA. Local TAWSS_mean_ [120.1 (101.0–233.7) vs. 75.5 (67.0–119.0) *10^−2^ (Pa), *p* = 0.048], OSI_mean_ [3.5 (2.3–6.2) vs. 0.8 (0.3–1.2)*10^−2^, *p* *=* 0.001], RRT_mean_ [288.1 (268.2–339.3) vs. 169.2 (143.9–248.1) *10^−2^ (Pa^−1^), *p* *=* 0.029], transWSS_mean_ [8.4 (6.9–15.9) vs. 3.8 (3.2–6.3)*10^−2^ (Pa), *p* *=* 0.008], CFI_mean_ [15.6 (12.9–20.2) vs. 8.5 (4.9–9.6)*10^−2^, *p* *=* 0.001) and TSVI_mean_ [148.8 (120.5–171.8) vs. 68.4 (44.2–86.6) (m^−1^), *p* < 0.001] were significantly higher in iCAE with thrombosis than in iCAE without thrombosis (Supplementary Table S3, Figures 3A–F). Furthermore, the comparison of the maximum values of the multidirectional WSS indicators between the two groups was generally consistent with the trends observed for the mean values, and the majority of these trends were statistically significant, except for that observed for TSVI (Supplementary Table S3, Supplementary Figures S3A–F). Instead, only TSVI_min_ of ectatic segments was significantly higher in ectatic RCA with thrombosis than in those without thrombosis [4.4 (3.4–7.4) vs. 2.7 (1.7–3.4) (m_−1_), *p* = 0.014] (Supplementary Table S3, Figures 4A–F). In addition, diffuse ectatic lesions were common in ectatic RCA (77.7%), and there were no significant differences between the two groups (75.0% vs. 78.6%, *p* *=* 1.000). The MVD in ectatic RCA with thrombosis was larger than that in ectatic RCA without thrombosis, but the difference did not meet the threshold of statistical significance [6.8 (5.0–8.9) vs. 5.0 (4.6–5.8) mm, *p* *=* 0.056] (Supplementary Table S3).

### 3.3 Multidirectional WSS metrics were independently associated with risk of thrombosis

The results of univariable and multivariable logistic regression analysis are presented in Table 2. Univariable analysis indicated that ectatic RCA, MVD, TAWSS_mean_, RRT_mean_, and transWSS_mean_ were associated with a higher risk of thrombosis in iCAE. Moreover, multivariable analysis showed that RRT_mean_ (OR = 1.010, 95% CI: 1.002–1.019, *p* *=* 0.021) and transWSS_mean_ (OR = 1.992, 95% CI: 1.257–2.939, *p* *=* 0.003) were independently associated with the thrombotic risk of iCAE.

**Table 2:** Univariable and multivariable logistic regression analysis of thrombotic events in iCAE.

Variables	Univariable analysis		Multivariable analysis	
	OR (95% CI)	*p* value	OR (95% CI)	*p* value
Ectatic RCA	5.333 (1.222–23.286)	0.026	10.082 (0.916–110.985)	0.059
Diffuse ectasia lesion	1.579 (0.298–8.563)	0.585	-	-
MVD (mm)	2.594 (1.349–4.988)	0.004	-	-
TAWSS_mean_ (*10^−2^) (Pa)	1.010 (1.001–1.020)	0.036	-	-
OSI_mean_ (*10^−2^)	0.995 (0.972–1.018	0.667	-	-
RRT_mean_ (*10^−2^) (Pa)	1.006 (1.000–1.012)	0.035	1.010 (1.002–1.019)	0.021
transWSS_mean_ (*10^−2^) (Pa)	1.753 (1.206–2.547)	0.003	1.992 (1.257–2.939)	0.003
CFI_mean_ (*10^−2^)	1.109 (1.000–1.230)	0.051	-	-
TSVI_mean_ (m^−1^)	1.001 (0.998–1.005)	0.532	-	-

RCA, right coronary artery; MVD, maximum vascular diameter; TAWSS, time-averaged wall shear stress; OSI, oscillatory shear index; RRT, relative residence time; transWSS, transverse wall shear stress; CFI, cross-flow index; TSVI, topological shear variation index.

### 3.4 Multidirectional WSS metrics can help stratify risk of thrombosis

ROC analysis revealed that transWSS_mean_ was the best single parameter to predict thrombotic events in iCAE (AUC = 0.885, sensitivity = 0.909, specificity = 0.881) (Supplementary Table S4). Besides, the AUC of OSI_mean_ (AUC = 0.868, sensitivity = 0.909, specificity = 0.786), CFI_mean_ (AUC = 0.864, sensitivity = 0.909, specificity = 0.810) and TSVI_mean_ (AUC = 0.861, sensitivity = 0.909, specificity = 0.810) were all higher than that of MVD (AUC = 0.789, sensitivity = 0.727, specificity = 0.810), although the differences were not statistically significant (Supplementary Table S4). The RRT_mean_ + transWSS_mean_ model achieved a greater AUC value than MVD, and the difference was statistically significant (0.911 vs. 0.789, *p* *=* 0.048) (Figure 4).

## 4 Discussion

In this study, we employed angiography-based three-dimensional reconstruction of coronary arteries, coupled with CFD analysis, to determine the effect of multidirectional WSS on the risk of thrombosis in iCAE. Our main findings were as follows: (i) multidirectional WSS metrics (including TAWSS_mean_, OSI_mean_, RRT_mean_, transWSS_mean_, CFI_mean_, and TSVI_mean_) of ectatic segments in iCAE with thrombosis were significantly higher than those of iCAE without thrombosis. The results of subgroup analysis for ectatic RCA were consistent; (ii) univariate and multivariate logistic regression analyses indicated that RRT_mean_ and transWSS_mean_ were independently associated with thrombotic events in iCAE; (iii) multidirectional WSS metrics (the RRT_mean_ + transWSS_mean_ model) showed a greater capacity for determining the risk of thrombosis in iCAE compared to the conventional measurement of MVD.

Compared to other aneurysmally ectatic blood vessels that are prone to rupture, the primary clinical concerns on iCAE are the risks of thrombosis and myocardial infarction (30). Numerous studies have indicated that the risk of thrombosis is not only associated with the MVD of the coronary artery but also is associated with hemodynamic alterations in the ectatic region. Sluggishness, stagnation, and blood flow turbulence have been implicated in thrombogenesis (1, 10, 11). Therefore, hemodynamic analysis may offer novel insights into iCAE and assist in the risk stratification of patients for appropriate medical intervention.

In previous hemodynamic studies, researchers have focused on a single CAA (11, 15). However, iCAE are more diffuse lesions (2). In clinical practice, a coronary artery can have several aneurysms or exhibit no distinct aneurysmal changes, instead showing a relatively uniform and diffuse ectasia (Figure 1). Focusing solely on single aneurysms can undermine the significance of hemodynamic data. Therefore, in this study, we considered the “entire” coronary artery as the primary subject of investigation to explore the relationship between its hemodynamic alterations and the risk of thrombosis. Moreover, since most studies have utilized only TAWSS, OSI, and RRT as indices of flow disturbance, they have not fully addressed the multidirectional nature of blood flow induced by its pulsatility in conjunction with the three-dimensional geometry (11, 31). Therefore, we introduced a broader array of WSS metrics to address this multidirectional flow behavior, including transWSS, CFI, and TSVI, as the first report of their application in CAE research.

Our study demonstrated significant hemodynamic differences between iCAE with thrombosis and iCAE without thrombosis. Consistent with the findings of Gutierrez *et al*. (15), iCAE with thrombosis exhibited markedly higher local RRT and OSI (mean value) compared to iCAE without thrombosis. Increased OSI and RRT are the primary characteristics of oscillatory and circulatory flow. They can induce the inflammatory response and platelet activation, thereby prmoting thrombogenesis (32). However, our results were different from those of Gutierrez et al. in terms of TAWSS. In our study, iCAE with thrombosis had higher TAWSS_mean_ and TAWSS_max_ but lower TAWSS_min_ compared to those without thrombosis. In contrast, Gutierrez et al. reported lower TAWSS in CAA with thrombosis (15). Despite differences, both studies reported lower TAWSS in the ectatic segments compared to adjacent normal segments (Figure 1). Possible reasons for this discrepancy may be: (i) the study populations were different between the two studies. Gutierrez *et al*. (15) focused on a single CAA, whereas the present study addressed iCAE, whose lesions tended to be more diffuse. Previous studies have confirmed the correlation between TAWSS distribution and the length of the coronary ectatic segment (32); (ii) the method of hemodynamic parameter acquisition varied between the two studies. Gutierrez et al. (15) did not mention how hemodynamic parameters were obtained in their study. In addition to TAWSS, RRT, and OSI, the novel hemodynamic parameters we introduced were significantly different between iCAE with thrombosis and iCAE without thrombosis. The results indicated that transWSS_mean_, CFI_mean_, and TSVI_mean_ were higher in iCAE with thrombosis than in iCAE without thrombosis. Increased transWSS_mean_, CFI_mean_, and TSVI_mean_ imply a greater magnitude of perpendicular shear force on the vessel wall by blood flow, increased lateral flow, a higher risk of turbulence, and more severe flow disturbance. These hemodynamic changes may further promote the formation of thrombosis (16–18). This hypothesis is supported by Russo et al. (33), who found that higher WSS in ruptured fibrous cap plaques correlated significantly with the expression of thrombosis-related genes: TNFα (R = 0.9, *p* < 0.001), MMP9 (*p* = 0.005, R = 0.7)—key mediators of inflammation and vascular integrity loss—and inversely with EDN1 (R = −0.4, *p* = 0.036), a vasoconstrictor gene. Although we did not measure gene expression in iCAE, the WSS-gene regulator*y* axis observed in ruptured fibrous cap plaques suggests iCAE's abnormal WSS may similarly modulate pro-thrombotic gene expression, a hypothesis requiring further validation.

Furthermore, our study was the first to identify an independent association between RRT_mean_ and transWSS_mean_ with the occurrence of thrombotic events in iCAE. Additionally, compared to MVD alone, the combination of hemodynamic parameters (AUC of RRT_mean_ + transWSS_mean_ model: 0.911) may better predict the risk of thrombosis in patients with iCAE (AUC of MVD: 0.789, *p* *=* 0.048). This finding may question the current clinical practice of relying on single MVD measurements to evaluate thrombosis risk and guide antithrombotic treatment decisions (8, 9). Moreever, Naturally, further validation through larger cohort studies is needed to confirm these observations. With the maturation and development of automated computational models, hemodynamic analysis can be easily and widely applied in clinical practice.

An additional phenomenon merits attention in the context of our study. In our study, RCA was the most frequently affected blood vessel in iCAE, with a prevalence of as high as 41.5%. These findings are largely consistent with those of previous reports (34, 35). This susceptibility was further supported by the findings of Wu et al. (36) who conducted fluid-structure interaction numerical simulations and demonstrated that the RCA is more susceptible to ectatic remodeling compared to the LAD and LCX. Additionally, our findings revealed a higher likelihood of RCA involvement in iCAE with thrombosis compared to iCAE without thrombosis (72.7% vs. 33.3%, *p* *=* 0.044). In univariate logistic regression analysis, ectatic RCA was correlated with a higher risk of thrombosis. However, subsequent multivariate analysis indicated that ectatic RCA was not an independent risk factor for thrombosis in iCAE (*p* *=* 0.059). Therefore, whether the unique hemodynamic characteristics of the RCA itself increase the risk of thrombosis in iCAE necessitates further studies.

There were some limitations to this study. First, all of the participants were from a single center, and the sample size was small; however, this study is currently the largest among CFD studies. Future multiple-center studies with larger sample sizes are needed to validate these findings. Second, ectatic RCA accounted for 41.5% of the total population and 72.7% of iCAE with thrombosis. Although this represents a typical distribution of iCAE treated in hospitals, it can bias the results. We conducted a subgroup analysis of ectatic RCA; however, our data volume was not large enough to separately analyze ectatic LAD and LCX, necessitating multiple-center studies with larger sample sizes. Third, similar to other retrospective studies, the hemodynamics of iCAE can be affected by the thrombotic event itself even in the presence of successful reperfusion therapy. Fourth, in our study, coronary branches <2 mm in diameter were not three-dimensionally reconstructed, and the consequent reduction in main branch blood flow was not fully incorporated into our analysis. Under such circumstances, the WSS in the main branch distal to these small branches may be overestimated. However, it must be emphasized that this limitation is also unavoidable in traditional CFD modeling due to the precision constraints inherent in three-dimensional coronary model reconstruction. Fifth, the current study did not account for the non-Newtonian behavior of blood, which may affect the accurate simulation of blood flow patterns in certain pathological conditions. However, this simplified design enables relatively accurate and rapid calculation of multidirectional WSS metrics, making the analysis clinically feasible. Sixth, our CFD simulations assumed rigid vessel walls with a no-slip boundary condition, which is not consistent with the dynamic scenario *in vivo* (37). Therefore, further refinement of the CFD method is necessary.

## 5 Conclusions

Local multidirectional WSS metrics (including TAWSS_mean_, OSI_mean_, RRT_mean_, transWSS_mean_, CFI_mean_, and TSVI_mean_) can help identify iCAE with a higher risk of thrombosis. RRT_mean_ and transWSS_mean_ are independently associated with thrombosis in iCAE. Multidirectional WSS metrics (the RRT_mean_ + transWSS_mean_ model) can help improve the stratification of the thrombosis risk in iCAE. Utilizing an automatic computational model, the hemodynamic analysis presents a promising clinical measure to assess the risk of thrombosis in iCAE.

## Data Availability

The original contributions presented in the study are included in the article/Supplementary Material, further inquiries can be directed to the corresponding authors.
