# Supplementary material for: Multidirectional wall shear stress is associated with thrombotic risk in isolated coronary artery ectasia
Source: Front Cardiovasc Med. 2025 Nov 18;12:1705263. doi: 10.3389/fcvm.2025.1705263 (PMC12669196; doi:10.3389/fcvm.2025.1705263)
Supplement: Supplementary file 1 [file Datasheet1.pdf]

Multidirectional Wall Shear Stress is Associated with Thrombotic Risk in Isolated Coronary Artery Ectasia

Jiejun Sun, MD,[1,2#] Muyun Tang, MD,[1,2 #] Shitong Liu, M.Eng,[3] Zhenyu Liu, MD,[1] Zhujun Shen, MD,[1] Hongzhi Xie, MD,[1] Wei Wu, MD,[1] Hao Qian, MD,[1] Liang Wang, MD,[1] Zhiyu Zhang, MD,[2] Ran Tian, MD,[1*] Shuyang Zhang, MD, [1,2*]

[1] Department of Cardiology, Peking Union Medical College Hospital, Chinese Academy of Medical Sciences & Peking Union Medical College, Beijing, China.

[2] Department of Medical Research Center, State Key Laboratory of Complex Severe and Rare Diseases, Peking Union Medical College Hospital, Chinese Academy of Medical Science and Peking Union Medical College, Beijing, China.

[3] Beijing Uninsim Tech Co. Ltd

[#]**Co-first author:** These authors share first authorship.

[*]**Corresponding author**

[*]**Ran Tian**

Address:1 Shuaifuyuan, Dongcheng District, Beijing,100730, China

Tel:+86 18501905148；Email: ron_tian@163.com

[*]**Shuyang zhang**

Address:1 Shuaifuyuan, Dongcheng District, Beijing,100730, China

Tel:+86 13535225012；Email: shuyangzhang103@nrdrs.org

Table S1. Baseline Clinical Characteristics of the Patients with iCAE

	Patients with iCAE (n=34)
Age, yrs	57.4±14.2
Male	25 (73.5)
Physical examination	
Body mass index (kg/m2)	26.3±4.6
Heart rate (beats/min)	74.8±11.5
Systolic pressure (mm Hg)	129.4±14.5
Diastolic pressure (mm Hg)	79.5±11.9
Cardiovascular risk factors	
Hypertension	28 (82.4)
Diabetes mellitus	4 (11.8)
Hypercholesterolemia	24 (70.6)
Reduced kidney function	0 (0)
Smoking	10 (29.4)
Drug regimen	
Antiplatelet drugs	0 (0)
Anticoagulant drugs	0 (0)
Biochemical markers	
Hematocrit (%)	40.7±3.4
Low-density lipoprotein cholesterol (mmol/L)	2.1±0.8
D-dimer (mg/L)	0.2 (0.2-0.4)
Echocardiogram parameters	
Left atrium diameter (mm)	39.3 ± 7.0
Right ventricular diameter (mm)	23.0 ± 5.0
Interventricular septum (mm)	9.0 (8.0-11.0)
Left ventricular posterior wall (mm)	8.0 (7.0-9.0)
Left ventricular end-diastolic diameter (mm)	49.2±5.0
Left ventricular ejection fraction (%)	67.0 (54.0-70.0)
Markis classificationa	
Type of I	12 (35.3)
Type of II	8 (23.5)
Type of III	9 (26.5)
Type of IV	5 (14.7)

iCAE=isolated coronary artery ectasia; [a]Markis classification is defined by Markis et.: Type I, diffuse ectasia of two or three vessels; Type II, diffuse disease in one vessel and localized disease in another vessel; Type III, diffuse ectasia of one vessel only; Type IV, localized or segmental ectasia.

Table S2. Calculation Formulas for Multidirectional Wall Shear Stress Metrics

Multidirectional wall shear stress metrics	Calculation formulas
Time averaged wall shear stress (TAWSS)	$\mathrm{TAWSS} = \frac{1}{\mathrm{T}}\int_0^{\mathrm{T}} \lvert\mathrm{WSS}\rvert\,\mathrm{dt}$
Oscillatory shear index (OSI)	$\mathrm{OSI} = 0.5\left[1\text{-}\left(\frac{\left\lvert\int_0^{\mathrm{T}}\mathrm{WSS}\,\mathrm{dt}\right\rvert}{\int_0^{\mathrm{T}}\lvert\mathrm{WSS}\rvert\,\mathrm{dt}}\right)\right]$
Relative residence time (RRT)	$\mathrm{RRT} = \frac{1}{\mathrm{TAWSS}\cdot(1\text{-}2\cdot\mathrm{OSI})}$
Transverse wall shear stress (transWSS)	$\mathrm{transWSS} = \frac{1}{\mathrm{T}}\int_0^{\mathrm{T}}\left\lvert\mathrm{WSS}\cdot\left(\mathrm{n}\times\frac{\int_0^{\mathrm{T}}\mathrm{WSS}\,\mathrm{dt}}{\left\lvert\int_0^{\mathrm{T}}\mathrm{WSS}\,\mathrm{dt}\right\rvert}\right)\right\rvert\mathrm{dt}$
Cross-flow index (CFI)	$\mathrm{CFI} = \frac{\mathrm{transWSS}}{\mathrm{TAWSS}}$
Topological shear variation index (TSVI)	$\mathrm{TSVI} = \left\{\frac{1}{\mathrm{T}}\int_0^{\mathrm{T}}\left[\nabla\cdot(\mathrm{WSS_u})\text{-}\overline{\nabla\cdot(\mathrm{WSS_u})}\right]^2\,\mathrm{dt}\right\}^{1/2}$

WSS is the time-varying wall shear stress vector; **T** is the cardiac cycle duration; **n** is the unit vector normal to the arterial surface at each element; **$\mathbf{WSS_u}$** is the **WSS** unit vector; $\nabla\cdot(\mathrm{WSS_u}) = \mathrm{DIV_{WSS}}$ is the divergence of the **WSS** unit vector field; the overbar denotes a cycle-average quantity.

Table S3. Imaging and Hemodynamic Characteristics of Ectatic RCA

		Ectatic RCA with thrombosis (n=8)	Ectatic RCA without thrombosis (n=14)	P value
Diffuse ectatic lesion[a]		6 (75.0)	11 (78.6)	1.000
MVD (mm)		6.8 (5.0-8.9)	5.0 (4.6-5.8)	0.056
Multidirectional shear stress metrics				
	Mean	120.1 (101.0-233.7)	75.5 (67.0-119.0)	0.048
TAWSS ($*10^{-2}$) (Pa)	Maximum	777.6 (286.3-1312.3)	250.5 (174.7-433.2)	0.017
	Minimum	10.2 (8.9-15.9)	15.1 (12.2-18.1)	0.076
	Mean	3.5 (2.3-6.2）	0.8 (0.3-1.2)	＜0.001
OSI ($*10^{-2}$)	Maximum	35.1 (24.4-40.9)	17.5 (5.8-28.9)	0.034
	Minimum	0.0025 (0.0005-0.0046)	0.0004 (0.0002-0.0016)	0.065
	Mean	288.1 (268.2-339.3)	169.2 (143.9-248.1)	0.029
RRT ($*10^{-2}$) (Pa^{-1})	Maximum	3838.9 (2226.1-5594.4)	1568.3 (827.6-3068.7)	0.035
	Minimum	14.5 (8.4-35.2)	40.7 (29.1-58.1)	0.012
	Mean	8.4 (6.9-15.9)	3.8 (3.2-6.3)	0.008
transWSS ($*10^{-2}$) (Pa)	Maximum	37.8 (22.7-68.4)	12.6 (10.7-18.2)	0.009
	Minimum	0.3 (0.2-1.1)	0.2 (0.1-0.5)	0.172
	Mean	15.6 (12.9-20.2)	8.5 (4.9-9.6)	＜0.001
CFI ($*10^{-2}$)	Maximum	65.4±3.4	48.3±22.0	0.020
	Minimum	0.6 (0.3-0.7)	0.3 (0.2-0.7)	0.172
	Mean	148.8 (120.5-171.8)	68.4 (44.2-86.6)	＜0.001
TSVI (m^{-1})	Maximum	1252.0 (971.1-1384.7)	1041.0 (749.7-1162.7)	0.056
	Minimum	4.4 (3.4-7.4)	2.7 (1.7-3.4)	0.014

iCAE=isolated coronary artery ectasia; MVD=maximum vascular diameter; TAWSS=time-averaged wall shear stress; OSI=oscillatory shear index; RRT=relative residence time; transWSS=transverse wall shear stress; CFI=cross-flow index; TSVI=topological shear variation index. [a]Diffuse ectatic lesion was defined as those in which the ectatic segment extends beyond one-third of the arterial length.

Table S4. Accuracy of Geometric and Hemodynamic Parameters for Predicting Thrombotic Events in iCAE

Variables	AUC	Youden's index	Cutoff	sensitivity	specificity
MVD	0.789	0.537	6.0 mm	0.727	0.810
$TAWSS_{mean}$	0.745	0.580	$97.0*10^{-2}$ Pa	0.818	0.762
OSI_{mean}	0.868	0.695	$1.7*10^{-2}$	0.909	0.786
RRT_{mean}	0.751	0.556	$253.9*10^{-2}$ Pa^{-1}	0.818	0.738
$transWSS_{mean}$	0.885	0.79	$6.2*10^{-2}$ Pa	0.909	0.881
CFI_{mean}	0.864	0.719	$10.9*10^{-2}$	0.909	0.810
$TSVI_{mean}$	0.861	0.719	106.0 m^{-1}	0.909	0.810

AUC=area under the receiver-operating characteristic curve; MVD=maximum vascular diameter; TAWSS=time-averaged wall shear stress; OSI=oscillatory shear index; RRT=relative residence time; transWSS=transverse wall shear stress; CFI=cross-flow index; TSVI=topological shear variation index.

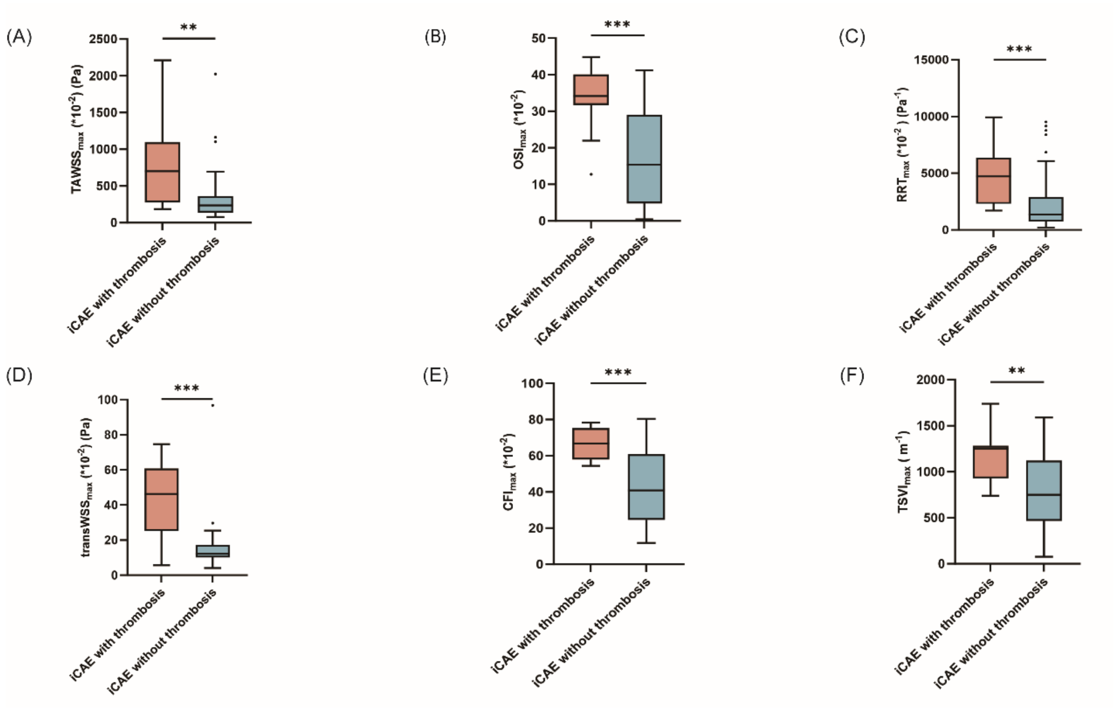

Figure S1. Comparison of the Maximum Values of Hemodynamic Parameters between iCAE with and without Thrombosis

*P < 0.05; **P < 0.01; ***P < 0.001. TAWSS=time-averaged wall shear stress; OSI=oscillatory shear index; RRT=relative residence time; transWSS=transverse wall shear stress; CFI=cross-flow index; TSVI=topological shear variation index.

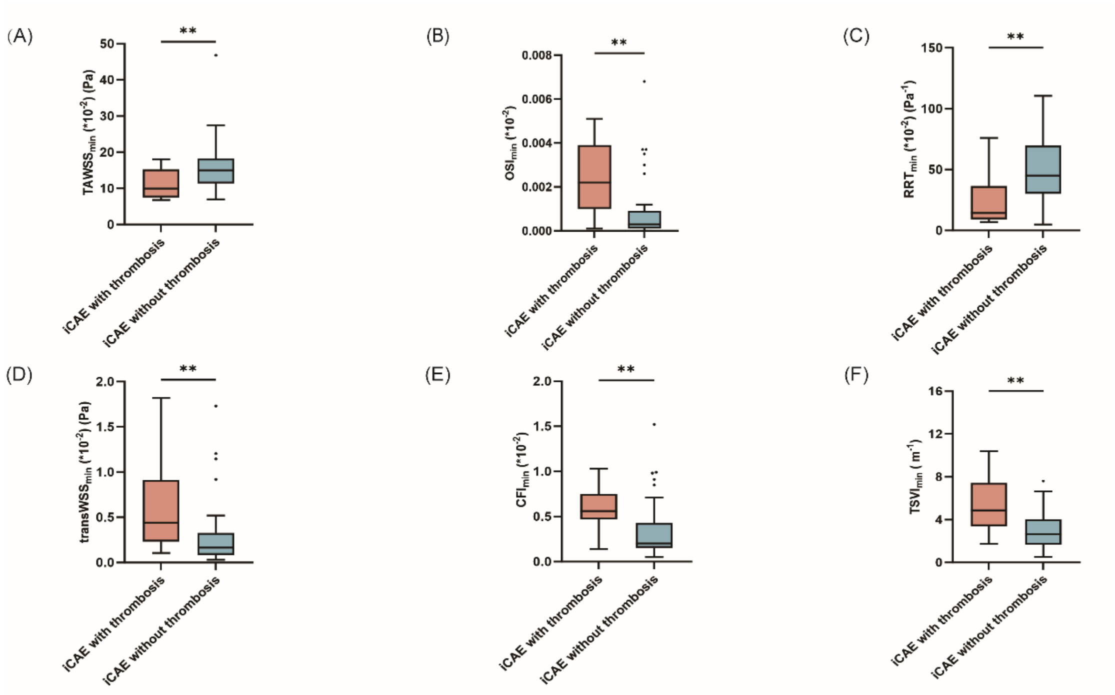

Figure S2. Comparison of the Minimum Values of Hemodynamic Parameters between iCAE with and without Thrombosis

*P < 0.05; **P < 0.01; ***P < 0.001. TAWSS=time-averaged wall shear stress; OSI=oscillatory shear index; RRT=relative residence time; transWSS=transverse wall shear stress; CFI=cross-flow index; TSVI=topological shear variation index.

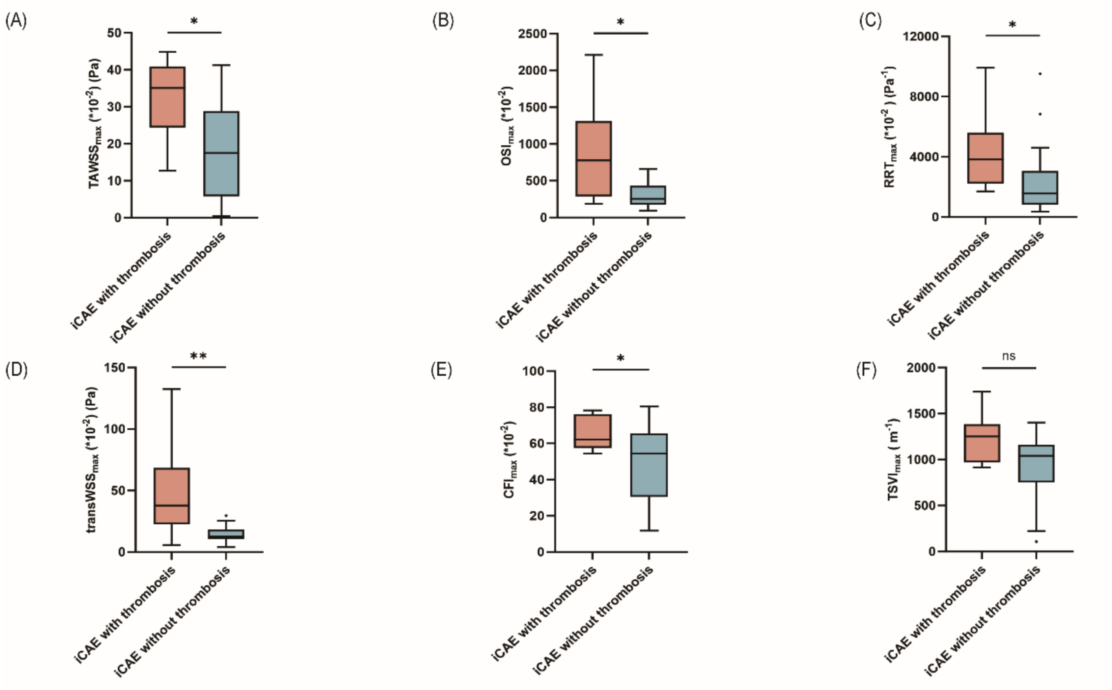

Figure S3. Comparison of the Maximum Values of Hemodynamic Parameters between Ectatic RCA with and without Thrombosis

*P < 0.05; **P < 0.01; ***P < 0.001. TAWSS=time-averaged wall shear stress; OSI=oscillatory shear index; RRT=relative residence time; transWSS=transverse wall shear stress; CFI=cross-flow index; TSVI=topological shear variation index.

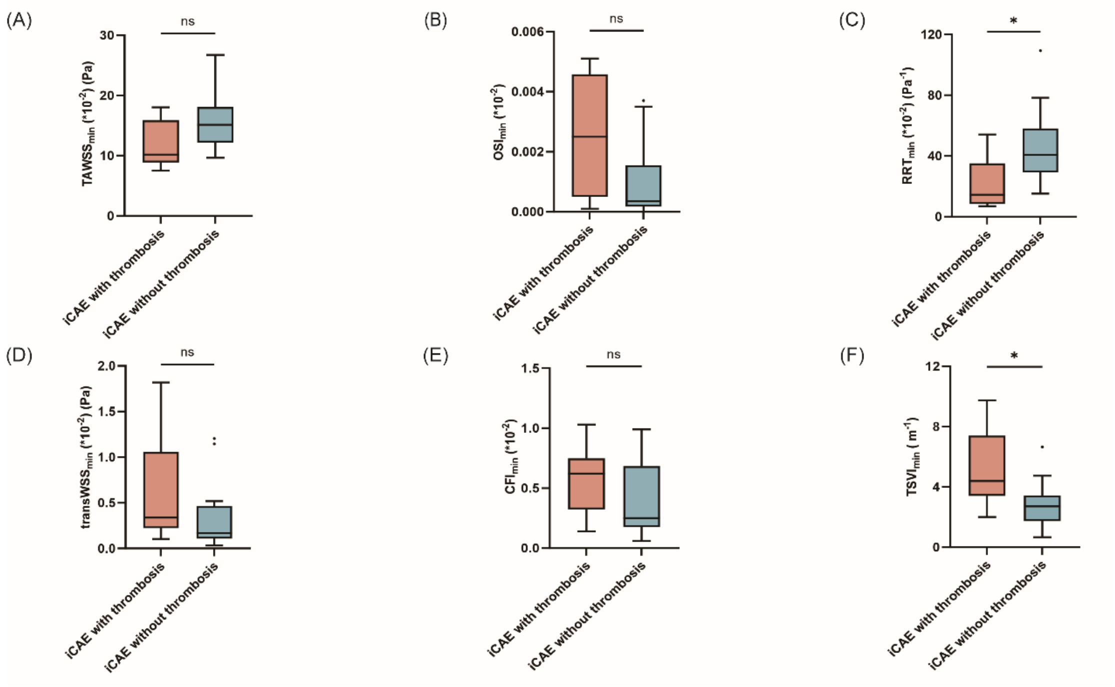

Figure S4. Comparison of the Minimum Values of Hemodynamic Parameters between Ectatic RCA with and without Thrombosis

*P < 0.05; **P < 0.01; ***P < 0.001. TAWSS=time-averaged wall shear stress; OSI=oscillatory shear index; RRT=relative residence time; transWSS=transverse wall shear stress; CFI=cross-flow index; TSVI=topological shear variation index.
